# Chronic Lymphocytic Thyroiditis Does Not Worsen Early Surgical Outcomes in Papillary Thyroid Cancer

**DOI:** 10.1002/wjs.70012

**Published:** 2025-07-28

**Authors:** Tanaz M. Vaghaiwalla, Jonathan Chang, Victoria DeTrolio, Cima Saghira, Mehmet Akcin, Cheng‐Bang Chen, John I. Lew

**Affiliations:** ^1^ Division of Endocrine Surgery DeWitt Daughtry Department of Surgery University of Miami Miller School of Medicine Miami Florida USA; ^2^ DeWitt Daughtry Department of Surgery University of Miami Miller School of Medicine Miami Florida USA; ^3^ Department of Industrial Systems Engineering University of Miami Miller School of Medicine Miami Florida USA

**Keywords:** autoimmune thyroid disease, Hashimoto's thyroiditis, thyroidectomy

## Abstract

**Background:**

Chronic lymphocytic thyroiditis (CLT), an autoimmune thyroid disorder that most commonly causes hypothyroidism in women, may confer a higher surgical risk for patients undergoing thyroidectomy for papillary thyroid carcinoma (PTC). This study evaluates surgical treatment outcomes for patients with a diagnosis of PTC with and without CLT.

**Methods:**

A retrospective review of prospectively collected data for patients who underwent thyroidectomy from 2009 to 2020 at a tertiary institution was performed. Patients ≥ 18 years of age were subdivided into 2 groups: patients with CLT and PTC and patients with PTC alone. Sociodemographic factors, tumor characteristics, final histopathology, thyroidectomy‐specific outcomes, and postoperative course were evaluated. Chi‐squared tests were used for categorical variables and comparisons based on *t*‐tests.

**Results:**

Of 1073 patients with PTC, most were women *n* = 872 (81%), Caucasian *n* = 933 (87%) with a mean of 48 (± 13) years of age, mean tumor size of 1.8 cm (± 1.3 cm), and low stage disease I/II *n* = 1049 (98%). Among patients with PTC *n* = 167 (16%) had a concurrent diagnosis of CLT. When comparing patients with PTC and CLT to PTC alone, there were no significant differences in age, race, or tumor size, respectively. When comparing patients with PTC and CLT to PTC alone, there were no significant differences in permanent recurrent laryngeal nerve injury (1.2% [*n* = 2] vs. 0.2% [*n* = 2]), bleeding and/or return to OR (0.6% [*n* = 1] vs. 0.7% [*n* = 6]), persistent hypocalcemia (0% [*n* = 0] vs. 0.33% [*n* = 3]), wound infection (0.6% [*n* = 1] vs. 0.4% [*n* = 4]), and radioactive iodine therapy (35.9% [*n* = 60] vs. 31.2% [*n* = 283]). Rates of lymph node positivity (26.9% [*n* = 45] vs. 30.1% [*n* = 273]), extrathyroidal extension (14.3% [*n* = 24] vs. 16.5% [*n* = 150]), and PTC recurrence (4.19% [*n* = 7] vs. 4.75% [*n* = 43]) were similar between groups.

**Conclusion:**

Of those undergoing total thyroidectomy for PTC, 16% of patients have concurrent underlying CLT. Underlying CLT is not associated with more aggressive tumor biology, higher rates of surgical complications, or PTC recurrence when performed by high‐volume thyroid surgeons.

## 1 Introduction

The annual incidence of thyroid cancer is rising, and this increase is almost exclusively due to papillary thyroid cancer (PTC) [1]. PTC is the most common endocrine malignancy in the United States and it is associated with excellent long‐term survival, often exceeding 97% at 20 years [2]. However, tumor characteristics associated with worsened outcomes include tumor size > 4 cm, age, extrathyroidal extension (ETE), aggressive features on histopathology, and regional or distant disease [1, 3, 4]. Current guidelines recommend total thyroidectomy and regional lymphadenectomy for high‐risk PTC with or without radioactive iodine ablation (RAI) treatment and thyroid stimulating hormone (TSH) suppression. In contrast, there are several management strategies for low‐risk PTC, which include thyroid lobectomy, active surveillance, and more recently, radiofrequency ablation [1, 5].

Chronic lymphocytic thyroiditis (CLT), also termed Hashimoto's Thyroiditis, is the most common cause of hypothyroidism affecting 22 per 100,000 individuals in the United States. The hallmark of CLT is autoimmune‐mediated failure of thyroid function with possible development of thyroid nodular disease [6]. CLT can be managed conservatively with thyroid hormone replacement in most cases; however, thyroidectomy may be indicated for persistent extrathyroidal symptoms, obstructive symptoms, and concurrent malignancy [7, 8, 9, 10]. CLT and PTC are found to coexist in 8%–36% of cases [11]. A relationship between CLT and PTC was originally described by Dailey et al. in 1955 [12]. Since then, no consensus has been reached on whether a causal association exists between CLT and the development of PTC or whether the coexistence of these diagnoses affects surgical and oncologic outcomes [6, 12, 13]. Although some studies suggest that CLT secondary to chronic inflammation confers a higher risk of PTC development, other studies suggest that thyroiditis may decrease tumor aggressiveness by stimulating antitumor immunity [6, 13, 14, 15].

Despite the frequency with which PTC and CLT coexist, the literature describing a different approach to surgical treatment for patients with PTC and concurrent CLT compared to patients with PTC alone remains scarce [14, 16]. Some factors contributing to a lack of consensus on management for PTC and concomitant CLT include the multiple criteria used to make the diagnosis of CLT, the varied guidelines available for low‐risk PTC, and small sample sizes and heterogeneous patient populations in previous studies [6, 11, 17]. This study examines tumor characteristics and surgical treatment outcomes for patients suffering from PTC with and without CLT at a high‐volume tertiary medical center.

## 2 Materials and Methods

A retrospective review of prospectively collected data for 1073 patients who underwent total thyroidectomy at a single tertiary medical center between 2009 and 2020 was performed. Adult patients ≥ 18 years of age who underwent total thyroidectomy for PTC based on the final histopathology were included in the analysis. Patients with an incidental PTC, defined as PTC outside of the index thyroid nodule based on the final histopathology, and papillary thyroid microcarcinoma (PTMC), defined as PTC < 1 cm, were also included in the analysis. Exclusion criteria included patients without a diagnosis of PTC on the final histopathology, prior thyroid surgery, a history of head/neck radiation therapy or exposure, lateral neck or metastatic disease, a history of Graves' disease, and incomplete medical records and data. Patients with follicular, medullary, or anaplastic thyroid carcinoma based on the final histopathology were also excluded.

All study patients underwent a preoperative evaluation with a high‐volume endocrine surgery service at a large tertiary medical center. The decision to perform total thyroidectomy for patients with PTC during the study period was multifactorial and included factors such as patient preference, multifocal bilateral nodular disease, symptomatology, and evolving practice patterns (e.g., American Thyroid Association [ATA] Guidelines). Medical history and physical examination, including signs and symptoms related to thyroid conditions, were obtained in all patients. Neck ultrasound and fine needle aspiration (FNA) biopsy of thyroid nodules were performed according to published ATA guidelines [1]. FNA cytology results were classified using the Bethesda System for Reporting Thyroid Cytopathology (BSRTC). Molecular testing was performed selectively for thyroid nodules with Bethesda III or IV cytology. Patients were subdivided into two cohorts based on final histopathology: (1) patients with PTC and a concurrent diagnosis of CLT and (2) patients with PTC alone without a concurrent diagnosis of CLT.

Demographic and preoperative clinical factors, intraoperative findings, and surgical outcomes were evaluated. Postoperative complications of interest included permanent recurrent laryngeal nerve (RLN) injury, bleeding and/or return to operating room (OR), transient hypocalcemia, persistent hypocalcemia, and wound infection. Transient recurrent laryngeal nerve (RLN) injury or vocal cord palsy was defined as voice changes or hoarseness lasting < 6 months after thyroidectomy, typically first noted between 10 and 14 days postoperatively. Such postoperative symptoms warranted referral to otolaryngology for confirmation of vocal cord palsy. Permanent RLN injury was defined as documented vocal cord paralysis, confirmed by an otolaryngologist using laryngoscopy at 6 months after the date of operation. Preoperative laryngoscopy was not routinely performed unless there was concern or a documented history of vocal cord dysfunction or persistent voice changes postoperatively. Patients underwent routine serum calcium monitoring postoperatively. If hypocalcemic, patients received calcium and/or vitamin D supplementation. Transient or temporary hypocalcemia was defined as low serum calcium levels < 8.5 mg/dL requiring oral calcium with or without vitamin D supplementation (i.e., calcitriol) for < 6 months, with resultant resolution to eucalcemia. Permanent hypoparathyroidism was defined as persistently low serum calcium levels < 8.5 mg/dL requiring continued oral calcium with or without vitamin D supplementation (i.e., calcitriol) at 6 months after the date of operation.

The tumor characteristics evaluated were thyroid nodule size, extrathyroidal extension, and central lymph node positivity. Central lymph node dissection was defined as the removal of level 6 lymph nodes and was performed selectively under two conditions: (1) when clinically indicated preoperatively according to ATA guidelines, and (2) when pathologic lymph nodes were encountered intraoperatively and confirmed by frozen section. Patients who underwent lateral neck dissection were not included in the study. Postoperative variables, which included radioactive iodine (RAI) therapy administration, follow‐up time (in months), recurrence, and survival, were also assessed. PTC recurrence was defined as evidence of either biochemical disease with elevated serum thyroglobulin levels or structural disease on imaging studies with subsequent confirmation by FNA of diseased tissue with elevated thyroglobulin washings at ≥ 6 months after surgical treatment. Patients with PTC underwent a postoperative visit 10–14 days after thyroidectomy, a 6‐month follow‐up, and annual visits for 5 years. Patients were established with endocrinologists and are concurrently followed for longer‐term cancer care.

Categorical data were analyzed using the *χ*2‐test or Fisher’s exact test. Continuous data were analyzed using unpaired *t*‐tests. Data were expressed as mean ± standard deviation for values with a normal distribution. Calculations were performed using a Statistical Package for the Social Sciences, Software Version 24.0 (IBM Corp, Armonk, NY). A *p* value of < 0.05 was considered statistically significant. This study was approved by the Institutional Review Board (IRB) at the University of Miami Leonard M. Miller School of Medicine.

## 3 Results

A total of 1073 patients who underwent total thyroidectomy were identified with a final histopathology of PTC. For the entire cohort of patients with PTC, 167 (16%) had a concurrent diagnosis of CLT on final histopathology. The majority of study patients were women (*n* = 872, 81%) with a mean age of 48 years (± 13 years). Mean follow‐up time for the entire cohort was 18 months. The median follow‐up was 12 months overall (Table 1). There was a significantly greater proportion of women compared to men in the cohort with PTC and CLT than PTC alone, (92.2% [*n* = 154] vs. 79.2% [*n* = 718] and *p* < 0.05), respectively. However, there were no significant differences for age between both subgroups (Table 2).

**TABLE 1:** Demographics and outcomes of total cohort.

Variable	Patients who underwent thyroidectomy for papillary thyroid cancer (*n* = 1073)
Sex	
Female	872 (81.3%)
Male	201 (18.7%)
Age, mean (std dev)	48.3 (13.3)
Radioactive iodine (RAI) therapy	343 (32.0%)
Tumor histopathology	
Tumor size	1.8 cm (1.3)
Extrathyroidal extension	174 (16.2%)
Lymph node positivity	318 (29.6%)
Surgical complications	
Transient vocal cord palsy	16 (1.5%)
Permanent RLN injury	4 (0.4%)
Bleeding/return to the OR	7 (0.7%)
Transient hypocalcemia < 6 Mo.	35 (3.3%)
Persistent hypocalcemia > 6 Mo.	3 (0.3%)
Wound infection	5 (0.5%)
Median follow‐up time	12 months
Recurrence	50 (4.7%)
Survival	1073 (100%)

Abbreviations: Ca, calcium; Mo, months; OR, operating room; RLN, recurrent laryngeal nerve.

**TABLE 2:** Demographics and outcomes of patient cohorts with PTC and CLT and with PTC alone.

Variable	PTC with CLT (*n* = 167)	PTC alone (*n* = 906)	*p*‐value
Sex			0.0001
Female	154 (92.2%)	718 (79.2%)	
Male	13 (7.8%)	188 (20.8%)	
Age, mean (std dev)	47.9 (12.7)	48.3 (13.5)	0.66
Radioactive iodine (RAI) therapy	60 (35.9%)	283 (31.2%)	0.27
Rate of central lymph node dissection	140 (83.8%)	722 (79.7%)	N.S.
Tumor histopathology			
Tumor size, mean (std dev)	1.7 cm (1.2)	1.8 cm (1.3)	0.52
Extrathyroidal extension	24 (14.4%)	150 (16.6%)	0.48
Lymph node positivity	45 (26.9%)	273 (30.1%)	0.19
Surgical complications			
Transient vocal cord palsy	4 (2.4%)	12 (1.3%)	0.48
Permanent RLN injury	2 (1.2%)	2 (0.2%)	0.23
Bleeding/return to the OR	1 (0.6%)	6 (0.7%)	N.S
Transient hypocalcemia < 6 Mo.	9 (5.4%)	26 (2.9%)	0.15
Persistent hypocalcemia > 6 Mo.	0 (0.0%)	3 (0.3%)	N.S.
Wound infection	1 (0.6%)	4 (0.4%)	N.S.
Median follow‐up time	12 months	12 months	N.S.
Recurrence	7 (4.2%)	43 (4.8%)	0.91
Survival	167 (100%)	906 (100%)	N.S.

Abbreviations: Ca, calcium; CLT, chronic lymphocytic thyroiditis; Mo, months; N.S., not significant; OR, operating room; PTC, papillary thyroid cancer; RLN, recurrent laryngeal nerve.

For the entire study group, the average tumor size was 1.8 cm (± 1.3 cm). Overall, 174 (16.2%) patients with PTC had ETE and 318 (29.6%) had lymph node positivity on the final histopathology. The majority of patients with PTC (*n* = 1049, 98%) had either stage I or stage II disease. After comparative analysis examining tumor histopathology for patients with PTC and CLT to those patients with PTC alone, there were no significant differences for mean tumor size (1.7 vs. 1.8 cm and *p* = 0.52), rates of ETE (14.3% [*n* = 24] vs. 16.5% [*n* = 150] and *p =* 0.48), or lymph node positivity (26.9% [*n* = 45] vs. 30.1% [*n* = 273] and *p =* 0.19), respectively (Table 2).

Complications after total thyroidectomy for the entire cohort included 16 (1.5%) patients with transient vocal cord palsy, 4 (0.4%) with permanent RLN injury, 7 (0.7%) with a bleeding event necessitating return to the OR, 35 (3.3%) with transient hypocalcemia, 3 (0.3%) with persistent hypocalcemia, and 5 (0.5%) with wound infection (Table 1). There were no significant differences between patients with PTC and CLT and patients with PTC alone for any of the thyroidectomy‐specific outcomes (Table 2).

A total of 343 (32.0%) patients received RAI therapy after thyroidectomy with no significant difference of its administration between patients with PTC and CLT and PTC alone (35.9% [*n* = 60] vs. 31.2% [*n* = 283] and *p =* 0.27), respectively (Table 2). When examining treatment outcomes for the entire cohort, PTC recurrence was identified in 50 (4.7%) patients at a mean follow‐up time of 18 months (Table 1). There was no difference in PTC recurrence for the two treatment groups (4.2% [*n* = 7] vs. 4.8% [*n* = 43] and *p* = 0.91), respectively (Table 2). No patients who underwent thyroidectomy in the study cohort experienced disease‐related death during the study period.

## 4 Discussion

CLT is an autoimmune thyroid disorder characterized by lymphocytic infiltration of the thyroid gland with destruction of thyroid follicular cells leading to hypothyroidism [18]. PTC is the most common type of thyroid malignancy and although it carries an excellent prognosis, its biology is diverse, ranging from indolent to metastatic disease with 15 distinct tumor variants. CLT is a common comorbidity in patients with PTC [11]. This coexistence, which has been documented as early as 1955, has been found to have implications on PTC tumor behavior and prognosis [12, 13]. Despite the number of studies since, there is still no consensus on how CLT affects prognosis and outcomes in patients with PTC [11, 15, 17].

The authors' study represents one of the largest surgical series examining tumor behavior and surgical outcomes in patients with PTC with and without CLT. Among all study patients with PTC, 16% had a concurrent diagnosis of CLT confirmed on final histopathology, which is consistent with the literature reporting a prevalence of 8%–36% [11]. Additionally, there was a marked female predominance in the cohort with PTC and CLT compared to PTC alone aligning with the known higher prevalence of both CLT and PTC in women [19]. When comparing patients with PTC and CLT to patients with PTC alone, there were no significant differences in the study variables spanning tumor behavior, surgical complications, or clinical outcomes.

The association of CLT in patients with PTC remains controversial. CLT is reported to play a paradoxical role in the development and progression of PTC, whereby lymphocytic infiltration facilitates initial tumor growth and indiscriminate autoimmune activity against both benign and malignant thyroid cells mitigates tumor aggressiveness [11, 20, 21, 22]. However, these associations are inconclusive with mixed findings across the literature. Some studies have shown that CLT may exert a protective effect against tumor progression, resulting in lower rates of lymph node positivity and ETE in individuals with concurrent CLT and PTC [12, 13, 19]. Contrary to these reports, the authors' current study identified no statistically significant differences in lymph node positivity or ETE between patients with PTC and CLT and patients with PTC alone. Tumor size was also similar between the study groups, which is consistent overall with the literature [19]. The authors' current study differs significantly from a prior retrospective study, which found CLT to be associated with smaller tumor size, higher rates of ETE, and lower rates of lymph node metastasis [17]. Differences in reported tumor behavior between this study and others may be explained, in part, by variations in the diagnosis of CLT, patient selection, environmental factors, and extent of thyroidectomy.

In the surgical treatment of PTC, studies suggest that CLT confers a higher perioperative risk due to enlargement of the thyroid gland and inflammation‐induced adhesions between the thyroid and surrounding soft tissues [23, 24]. Transient hypocalcemia, the most common surgical complication following total thyroidectomy, occurs in 20%–30% of all cases whereas permanent hypocalcemia, or persistently abnormal calcium levels > 6 months post‐thyroidectomy, occurs at a rate of 1.3%–3% for all patients. The authors' study reveals that CLT has little impact on the rates of both transient and persistent hypocalcemia among patients with PTC and CLT versus patients with PTC alone, with rates of 5.4% and 2.9% for transient hypocalcemia and 0% and 0.3% for permanent hypocalcemia, respectively. These findings align with a number of studies in the literature, which report no significant differences in either transient or persistent hypocalcemia rates between patients suffering from PTC with and without CLT, whereas other reports suggest statistically significant variations in transient hypocalcemia [24, 25, 26]. Rates of transient vocal cord palsy in the study population were 1.5% and 2.4% for the PTC and CLT cohort and PTC alone cohort, respectively, and such findings are similar to previous studies [24, 26]. Additionally, patients with PTC and CLT versus PTC alone in the authors' study had a permanent RLN injury at 1.2% and 0.2%, respectively, with all cases being unilateral, whereas reported rates of 0.3%–6% are found in the literature. Furthermore, these findings are consistent with prior reports, which showed no significant differences in RLN injury between patients with and without CLT after thyroidectomy for PTC [20, 24, 26, 27]. Other perioperative complications, such as bleeding events and/or return to the OR and wound infection, also showed no significant differences between the study cohorts aligning with literature reports of similarly low incidences [20, 24]. These results suggest that although CLT may introduce surgical challenges, its presence does not significantly alter the rates of perioperative complications in patients undergoing total thyroidectomy for PTC.

Coexisting CLT also appears to have little effect on RAI therapy, tumor recurrence, and survival with no significant differences observed for each respective variable between patients with PTC and CLT and patients with PTC alone. These results are in contrast to others in the literature, which found that RAI therapy was performed less in PTC patients with CLT; however, CLT was not found to be a prognostic factor for tumor recurrence [17]. Many studies present conflicting results with regard to the effect of CLT on PTC prognosis with most falling into two ideological camps—CLT has no effect on recurrence and CLT is associated with lower rates of recurrence. This discrepancy can be due to study differences in duration of follow‐up, diagnostic criteria, and cohort variability [13, 15, 28, 29, 30, 31]. Finally, despite the presence of CLT, total thyroidectomy for the management of PTC remains a safe and effective treatment with low morbidity.

Findings of the authors' study may contribute to future management recommendations for patients with concurrent PTC and CLT. There are no specific recommendations in current management guidelines regarding patients presenting with PTC and CLT, leaving a gap for evidence‐based decision‐making [1]. The authors' study suggests total thyroidectomy in addition to adjuvant RAI therapy between both patients with PTC and CLT and patients with PTC alone yields comparable surgical treatment outcomes. Although this may instill greater confidence in surgeons performing thyroidectomies in patients suffering from PTC with concurrent CLT, continued attention to the careful identification and preservation of the parathyroid glands is warranted as CLT‐related inflammation may disrupt anatomy and increase the risk of postoperative hypocalcemia. Accordingly, surgeons should maintain a low threshold for suspicion of hypocalcemia and RLN injury in patients with PTC and CLT after total thyroidectomy. These findings on the safety of thyroidectomy and the minimal impact of CLT on tumor behavior and recurrence can guide future guidelines to address clinical management gaps for this important PTC subgroup.

This study has several limitations inherent to a retrospective study design including referral and selection biases. TNM and ATA stages were not available for all patients and therefore not included. In terms of follow‐up time, long‐term follow‐up data were not available for all patients and thus were not included in the analysis. Certain disease‐specific factors, such as body mass index (BMI), thyroid gland weight, and retrosternal extension, were also not readily available and could not be assessed. Despite these limitations, worsened surgical outcomes were not observed between the two cohorts. All patients were treated at a high‐volume center by fellowship trained endocrine surgeons, and therefore, the study results may not be generalizable to all medical centers. Additionally, the study may have been underpowered to detect a difference between the study groups. Multicenter and prospective studies may be needed to validate the study findings.

In conclusion, patients with PTC and concurrent CLT do not experience worse treatment outcomes compared to patients with PTC alone, when thyroidectomy is performed by high‐volume experienced thyroid surgeons. Additionally, patients with PTC and concurrent CLT do not exhibit higher rates of aggressive tumor characteristics than patients with PTC alone. Underlying CLT is not associated with higher rates of PTC disease recurrence and optimal clinical outcomes are achieved in the presence or absence of concurrent CLT. The authors' findings identify a potential gap that exists in current guidelines for PTC management in the setting of concurrent CLT. Despite the frequency with which both conditions may exist, the authors' study demonstrates that total thyroidectomy is a safe operation with optimal early clinical outcomes when performed by high‐volume thyroid surgeons. Limited reports address the impact of CLT on PTC in the literature, and the authors' large surgical series underscores the clinical relevance of CLT for thyroid surgeons treating patients with PTC in their practice.

## Author Contributions

**Tanaz M. Vaghaiwalla:** conceptualization, data curation, formal analysis, investigation, methodology, validation, writing – original draft, writing – review and editing, visualization, supervision, project management. **Jonathan Chang:** writing – original draft, supervision, writing – review and editing. **Victoria DeTrolio:** data curation, writing – original draft. **Cima Saghira:** data curation, methodology, visualization. **Mehmet Akcin:** investigation, data curation, methodology. **Cheng‐Bang Chen:** investigation, methodology, formal analysis, validation, visualization. **John I. Lew:** conceptualization, methodology, writing – review and editing, visualization, supervision, project management.

## Conflicts of Interest

The authors declare no conflicts of interest.

## Data Availability

The data that support the findings of this study are available from the corresponding author upon reasonable request.
